# Willin/FRMD6 Mediates Mitochondrial Dysfunction Relevant to Neuronal Aβ Toxicity

**DOI:** 10.3390/cells11193140

**Published:** 2022-10-06

**Authors:** Doris Chen, Wanjia Yu, Laura Aitken, Frank Gunn-Moore

**Affiliations:** School of Biology, University of St Andrews, St Andrews KY16 9TF, UK

**Keywords:** Willin/FRMD6, mitochondrial dysfunction, oxidative stress, Alzheimer’s disease, neurodegeneration, ERK signaling

## Abstract

Willin/*FRMD6* has been reported as a potential Alzheimer’s disease (AD) risk gene in a series of genome-wide association and neuroimaging studies; however, the mechanisms underlying its potential role in AD pathogenesis remain unknown. Here, we demonstrate the direct effects of Aβ on Willin/FRMD6 expression and position mitochondrial oxidative stress as a novel potential mechanism underlying the role of Willin/FRMD6 in AD pathogenesis. Specifically, using mouse hippocampal HT-22 cells and primary mouse neurons, we show that Aβ induces downregulation of Willin/FRMD6 protein expression. Furthermore, we demonstrate that Willin/FRMD6 knockdown leads to mitochondrial dysfunction and fragmentation, as well as upregulation of ERK1/2 signaling, both of which are reported to be key early features of AD pathogenesis. Importantly, increasing Willin/FRMD6 expression was able to rescue Aβ-induced abnormalities in mitochondrial morphology, function, and energetics. Thus, enhancing Willin/FRMD6 expression holds potential as a therapeutic strategy for protecting against Aβ-induced mitochondrial and neuronal dysfunction.

## 1. Introduction

Alzheimer’s disease (AD) is the most common form of dementia, affecting more than 6 million Americans over the age of 65 (www.alz.org, accessed on 1 August 2022). Accumulation of amyloid beta (Aβ) peptides has long been implicated in the pathogenesis of AD; however, the underlying mechanisms are not well understood. Neurons, with their uniquely polarized structures and high energy demands, are highly dependent on proper mitochondrial function to provide the energy needed for maintenance of neuronal activity and survival [1]. Thus, key early pathological events in AD include mitochondrial dysfunction leading to the production and accumulation of excessive reactive oxygen species that impair neuronal function many years prior to the appearance of symptoms and pathological hallmarks such as senile plaques and neurofibrillary tangles [2].

Given the high estimated heritability of late-onset AD (LOAD) (~60–80%) [3,4], genome-wide association studies (GWAS) have been utilized in the AD field to identify causal variants and genes linked to AD susceptibility. GWAS studies and AD neuroimaging studies have identified several variants within the gene for 4.1-ezrin-radixin-moesin (FERM) domain-containing protein 6 (FRMD6), also known as Willin, that are associated with AD risk and hippocampal atrophy [5,6,7,8]. Since the initial discovery of Willin/FRMD6 as a novel binding partner of neurofascin 155 in rat sciatic nerves [9], studies have shown that functions of Willin/FRMD6 in neuronal cells include peripheral nerve repair [10], neuropeptide exocytosis [11], and neuronal differentiation [12]. Additionally, it has recently been shown that Willin/FRMD6 plays a role in the upstream regulation of both Hippo and ERK signaling pathways through which it influences cell proliferation and mechanical phenotype by modulation of the actin cytoskeleton [12,13,14,15]. Though activation of Hippo and ERK signaling pathways have been associated with neuronal death in neurodegeneration and AD [16,17,18], the role of upstream signaling components such as Willin/FRMD6 remains unclear.

Furthermore, while there is evidence for Willin/*FRMD6* transcript downregulation in microarray data from AD mouse models [19], the mechanisms linking Willin/FRMD6 to AD pathogenesis remain elusive. Given the involvement of Willin/FRMD6 in cellular functions such as ERK signaling and cytoskeletal organization that are critical in the maintenance of mitochondrial homeostasis and the role of mitochondrial dysfunction in early AD pathogenesis, we hypothesize that oxidative stress and alterations in mitochondrial function represent a mechanism linking Willin/FRMD6 to AD pathogenesis. In this study, we determined whether and how Willin/FRMD6 protein expression is altered by Aβ. Next, using both mouse hippocampal HT-22 cells and primary mouse neurons, we comprehensively evaluated changes in mitochondrial morphology and function resulting from knockdown of Willin/FRMD6. HT-22 cells are immortalized mouse hippocampal neurons that have been widely used as in vitro models to investigate mitochondrial alterations and dysfunction in the context of both amyloid [20,21] and tau [22] pathology relevant to AD pathogenesis. Furthermore, we address whether restoring Willin/FRMD6 expression can ameliorate Aβ-induced mitochondrial dysfunction. These studies provide the first direct evidence that Willin/FRMD6 is involved in the regulation of mitochondrial morphology and function and that Aβ and oxidative stress lead to altered Willin/FRMD6 levels in neuronal cells.

## 2. Materials and Methods

### 2.1. Analysis of Microarray and RNA-Seq Data from AD Mouse Models and AD Patient Brains

Previously obtained microarray data using total RNA prepared from human medial temporal gyrus (16 AD, 12 non-AD cases, accession number GSE5281 [23]) and 8 month old mouse hippocampi (3 HO [*SwAPP* K670N/M671L, *PSEN1* M146V], 10 wildtype, accession number GSE64398 [24]) were obtained from the GEO database and analyzed using GEO2R (www.ncbi.nlm.nih.gov/geo/geo2r/, accessed on 8 September 2022).

Previously obtained RNA-Seq data using total RNA from human brains (3 AD, 3 non-AD controls [pooled from 33 individuals], accession number PRJNA79871) were obtained from the European Nucleotide Archive. Raw read files were quality checked using *FastQC* (v0.11.8, Babraham Bioinformatics, Cambridge, UK) [25]. Trimming (quality threshold 20, minimum sequence length 20) was performed using *Flexbar* (v3.4.0, Berlin Institute for Medical System Biology, Berlin, Germany) [26]. Trimmed sequences were aligned to *hg38* (GRCh38) reference human genome using *HISAT2* (v2.1.0, University of Texas Southwestern Medical Center, Dallas, TX, USA) [27]. Gene expression quantification was performed using *featureCounts* (v2.0.1, The University of Melbourne, Parkville, Australia) [28] with multimapping reads not counted. Read counts were analyzed for differential expression using *DESeq2* (v1.34.0, European Molecular Biology Laboratory, Heidelberg, Germany) [29].

RNA-seq data for Willin/*FRMD6* from three AD mouse models (3 TPM [*PSEN1* M146V], 4 TAU [*MAPT* P301L], and 4 HO [*SwAPP* K670N/M671L, *PSEN1* M146V], 8 wildtype) at 8 months old were downloaded from the Mouseac online database (www.mouseac.org, accessed on 8 September 2022) [30] and analyzed as previously described [31]. Briefly, the gene expression values were log transformed and tested for statistical significance by fitting a linear regression model (Gene expression ~ Genotype).

### 2.2. Cell Lines

Mouse hippocampal HT-22 cells were purchased from EMD Millipore (La Jolla, CA, USA). Cells were cultured in DMEM (Gibco, Paisley, UK) containing 10% fetal bovine serum (Biowest, Nuaillé, France) and 1× Penicillin-Streptomycin (Gibco, Grand Island, NY, USA). Cells were maintained in a humidified 37 °C, 5% CO_2_ incubator.

### 2.3. Generation of Stable HT-22 Cells with Overexpression and Knockdown of Willin/FRMD6

Short hairpin RNAs (shRNA) designed to knockdown mouse Willin/*Frmd6* (21-mer target sequences: *Willin A*, 5′-CGAATTGCAAGATACTATTAT-3′, *Willin B*, 5′-CAAGAGTGTCTTTCTATTCAT-3′) were synthesized and cloned into pLKO.1puro (Addgene, plasmid 8453). Scrambled control, *shScr,* was obtained from Addgene (plasmid 1864). To generate lentiviral particles for knockdown of Willin/FRMD6 expression, HEK 293T cells were transfected with pLKO.1puro constructs along with packaging vector psPAX2 (Addgene plasmid 12260) and envelope vector pMD2.G (Addgene plasmid 12259) using TransIT^®^-LT1 (Mirus, Madison, WI, USA). Culture media was harvested at 48 and 72 h post-transfection. To generate retroviral particles for overexpression of Willin/FRMD6, Phoenix A cells were transfected with pBabepuro-Willin or pBabepuro (vector) (described in [32]) using TransIT^®^-LT1. Culture media was harvested at 48 and 72 h post-transfection.

Stable cell lines were generated using mouse hippocampal HT-22 cells. 24 h prior to transduction, HT-22 cells were plated at 10^5^ cells/cm^2^. Viral particles were added to cells with the addition of 8 μg/mL polybrene. 48 h post-transduction, cells were selected using 5 μg/mL puromycin (Sigma-Aldrich, St. Louis, MO, USA). For overexpression, monoclonal selection was performed by limiting dilution. Stable cells were maintained in culture media supplemented with 5 μg/mL puromycin. Successful knockdown or overexpression was confirmed by immunoblot.

### 2.4. Primary Neuronal Culture

Animal studies were approved by the School of Biology Ethics Committee at the University of St Andrews. C57BL/6 mice were bred as needed. Mouse primary hippocampal and cortical neurons were cultured from postnatal Day 0–1 pups. Brains were dissected in cold DMEM and dissociated with 0.05% Trypsin (Gibco, Paisley, UK) at 37 °C for 15 min prior to trituration. Cortical neurons were passed through a 40 μm cell strainer (Corning Falcon, Durham, NC, USA) and centrifuged for 5 min at 180× *g*. The pellet was resuspended in neuron culture medium (Neurobasal A (Gibco, Grand Island, NY, USA) supplemented with 1× B-27 (Gibco, Grand Island, NY, USA), 1× GlutaMAX (Gibco, Paisley, UK), 1× Penicillin-Streptomycin) and seeded on poly-D-lysine (Gibco, Carlsbad, CA, USA) coated culture plates (ThermoFisher Scientific, Rochester, NY, USA), coverslips (VWR, Lutterworth, UK), or Lab-Tek chambered coverglasses (ThermoFisher Scientific, Rochester, NY, USA) at appropriate densities for the intended studies. To enrich the culture for neurons and suppress glial cell proliferation, neurons were treated with 1 μM 5-Fluoro-2′deoxyuridine (FdU, Cayman Chemical, Ann Arbor, Michigan) on DIV 4. Neurons were transduced on DIV 6–8. Neurons were cultured for 10–21 days and treated as indicated. Aβ treatment was performed in Neurobasal A supplemented with 0.5× B-27 as previously described [33].

### 2.5. Adeno-Associated Virus Production

The human Willin/*FRMD6* ORF was subcloned into a pAAV vector backbone under the influence of the hSynapsin promoter (Addgene plasmid 51697) using EcoRI and BamHI restriction enzymes. An empty vector was produced by blunt-end ligation of the pAAV vector following EcoRI and BamHI digestion. HEK 293T cells were triple-transfected with AAV construct, AAV packaging plasmid pAAV2/1 (Addgene plasmid 112862), and AAV helper plasmid pAdDeltaF6 (Addgene plasmid 112867), using TransIT^®^-LT1. 24 h post-transfection, medium was replaced with fresh DMEM. Culture media and cells were collected 72 h post-transfection. AAV was harvested by three freeze–thaw cycles between a dry ice ethanol bath and a 37 °C water bath. Viral titer was determined by qRT-PCR.

### 2.6. Aβ Oligomer Preparation

Aβ oligomers were prepared as previously described [34,35]. Briefly, lyophilized Aβ peptide (GenicBio, Shanghai, China) was treated with HFIP (Sigma-Aldrich, St. Louis, MO, USA) to ensure monomerization. HFIP was removed by evaporation and pellets were resuspended to 5 mM in DMSO and bath sonicated for 15 min. The DMSO solution was diluted to 200 μM in DPBS (Gibco, Paisley, UK), vortexed for 30 s, and incubated for 24 h at 4 °C. Formation of oligomeric Aβ was confirmed through immunoblotting.

### 2.7. HT-22 Cell Treatments

HT-22 cells were plated to be 70–90% confluent at 24–72 h post-seeding before exposure to Aβ oligomers in serum-free media. For mitoTEMPO (Sigma-Aldrich, St. Louis, MO, USA) pre-treatment, HT-22 cells were pre-treated with mitoTEMPO for 1 h at the indicated concentrations prior to exposure to Aβ.

### 2.8. Immunoblotting

Cells were washed twice with PBS and incubated for 5 min on ice with Cell Lysis Buffer (Cell Signaling Technology, Danvers, MA, USA) supplemented with protease inhibitor (Roche, Mannheim, Germany) and 1 mM phenylmethylsulfonylfluoride (PMSF) (Sigma-Aldrich, St. Louis, MO, USA). Cells were transferred to microcentrifuge tubes and incubated for 30 min with agitation at 4 °C. Extracts were centrifuged at 3800× *g* for 10 min at 4 °C and the supernatants stored at −80 °C. Protein concentration was determined by BCA assay (Pierce, Rockford, IL, USA). Proteins were resolved by SDS-PAGE and electroblotted onto a 0.45 μm nitrocellulose membrane (GE Healthcare, Uppsala, Sweden). Non-specific binding was blocked using 5% skim milk (Marvel, St Albans, UK) or 5% FBS (Fisher Scientific, Loughborough, UK) in TBS. Membranes were probed with the following primary antibodies: rabbit anti-Willin/FRMD6 (1:1000, Cell Signaling Technology, Danvers, MA, USA), rabbit anti-phospho ERK (1:1000, Cell Signaling Technology, Danvers, MA, USA), mouse anti-ERK1/2 (1:1000, Cell Signaling Technology, Danvers, MA, USA), rabbit anti-phospho-DRP1-S616 (1:1000, Cell Signaling Technology, Danvers, MA, USA), rabbit anti-DRP1 (1:1000, Cell Signaling Technology, Danvers, MA, USA), rabbit anti-MAP2 (1:1000, Cell Signaling Technology, Danvers, MA, USA), rabbit anti-OPA1 (1:1000, Cell Signaling Technology, Danvers, MA, USA), rabbit anti-MFF (1:1000, Cell Signaling Technology, Danvers, MA, USA), mouse anti-β-actin (1:10,000, Sigma-Aldrich, St. Louis, MO, USA), mouse anti-GAPDH (1:10,000, Sigma-Aldrich, St. Louis, MO, USA). Blots were incubated with secondary antibodies conjugated to horse radish peroxidase (1:10,000, Abcam, Cambridge, UK). Immunoreactive bands were detected by enhanced chemiluminescence (Pierce, Rockford, IL, USA) using a Fujifilm LAS-3000 Imager (Tokyo, Japan). Band intensity was quantified using ImageLab (v6.1.0, Bio-Rad, Hercules, CA, USA).

### 2.9. Measurement of Mitochondrial Membrane Potential and Mitochondrial Reactive Oxygen Species

Cells were plated at low density on Lab-Tek eight-well chamber slides (for imaging) or black-walled 96-well plates (for spectrophotometry) and stained with 20 nM TMRM (non-quench mode) (Invitrogen, Eugene, OR, USA) in growth media at 37 °C for 30 min or 2.5 μM MitoSOX^TM^ Red (Invitrogen, Eugene, OR, USA) in HBSS for 20 min. Live cells were either imaged using a Leica TCS SP8 confocal microscope (Mannheim, Germany) using a 63× oil immersion objective or fluorescence intensity was determined using a SpectraMaxM2e plate reader (Molecular Devices, San Jose, CA, USA).

### 2.10. Mitochondrial Function Assays

Cytochrome c oxidase activity was measured in cell lysates as described previously [36,37]. Briefly, cell lysate was added to a cuvette containing 950 μL of assay buffer (10 mM Tris-HCl, pH 7 with 120 mM KCl) and the reaction volume brought to 1050 μL with enzyme dilution buffer (10 mM Tris-HCl, pH 7.0 containing 250 mM sucrose). The reaction was started by addition of 50 μL of ferrocytochrome c substrate solution (0.22 mM) and change in absorbance at 550 nm was measured at 10 s intervals for 3 min using a SpectraMaxM2e spectrophotometer.

ATP levels were measured with the ATP Bioluminescence Assay HS II Kit (Roche, Mannheim, Germany) according to the manufacturer’s instructions. Briefly, cells were washed twice with cold PBS before addition of ATP Lysis Buffer. Cells were harvested, incubated on ice for 30 min, and then centrifuged at 12,000× g. Protein content in the supernatant was determined by BCA assay and equal amounts of sample were added to a 96-well plate. The reaction volume was brought to 50 μL/well with ATP Dilution Buffer and ATP levels were determined by a CLARIOstar Plus microplate reader (BMG LabTech, Aylesbury, UK).

Total H_2_O_2_ levels in cell lysates were measured using the Amplex^TM^ Red Hydrogen Peroxide/Peroxidase Assay Kit (Invitrogen, Eugene, OR, USA) according to the manufacturer’s instructions using a SpectraMaxM2e spectrophotometer.

To assess mitochondrial redox activity, cells were incubated with MTT (3-(4,5-dimethylthiazol-2-yl)-2,5-diphenyltetrazolium bromide) dye solution at a final concentration of 0.5 mg/mL for 2–4 h. Afterwards, solubilization solution (4 mM HCl, 0.1% NP40 in isopropanol) was added to stop the reaction and absorbance was read at 570 nm with a reference wavelength of 690 nm using a SpectraMaxM2e spectrophotometer. 

### 2.11. Immunofluorescence

Cells were grown at low density on coverslips. For visualization of mitochondrial morphology, cells were incubated with 200 nM MitoTracker ^TM^ Red CMXRos (Invitrogen, Eugene, OR) for 20 min. Cells were fixed in cold 4% paraformaldehyde and permeabilized and blocked with 0.3% Triton X-100 and 5% goat serum in PBS for 1 h at room temperature. Cells were incubated with the following primary antibodies diluted in 5% goat serum in PBS at 4 °C overnight: rabbit anti-TOM20 (1:300, Cell Signaling Technology, Danvers, MA, USA), chicken anti-MAP2 (1:1000, Invitrogen, Eugene, OR, USA), rabbit anti-Willin/FRMD6 (1:100, Cell Signaling Technology, Danvers, MA, USA). Corresponding fluorescent secondary antibodies (Alexa Fluor^TM^ 488, 568, 594, 647, 1:1000, Invitrogen, Eugene, OR, USA), were diluted in blocking buffer and incubated for 1 h at room temperature. Coverslips were mounted in ProLong^TM^ Diamond Antifade Mountant (Invitrogen, Eugene, OR, USA) and imaged with a Leica TCS SP8 confocal microscope using a 63× oil immersion objective or a Leica DM5500B epifluorescence microscope (Mannheim, Germany) using a 40× oil immersion objective.

### 2.12. Quantification of Mitochondrial Morphology and Networks

Post-acquisition processing and analysis was performed with NIH ImageJ (v1.53q, National Institutes of Health, Bethesda, MD, USA) (primary neurons) or MitoSegNet (German Research Center for Environmental Health, Neuherberg, Germany) [38] (HT-22 cells) to assess mitochondrial morphology, network parameters, and fluorescence intensity. Numerical codes were used to blind the investigator to the experimental groups.

### 2.13. Statistical Analysis

Data in bar charts are presented as mean +/− SEM. Statistical analysis was performed using R (v4.1.1, Foundation for Statistical Computing, Vienna, Austria). Normality was assessed by Shapiro–Wilk test. Normally distributed data were analyzed using parametric Student’s *t* tests, Dunnett’s tests, or one-way ANOVA test with Tukey post hoc test, as appropriate. Non-normally distributed data were analyzed by non-parametric Mann–Whitney U test or Kruskal–Wallis tests. *p* < 0.05 was considered significant.

## 3. Results

### 3.1. Willin/FRMD6 Transcripts Are Downregulated in AD Mouse Models and AD Patient Brains

Previous microarray studies have demonstrated significant reduction of Willin/*Frmd6* transcripts in the cortices of APP^NL-G-F/NL-G-F^ and 3×Tg-AD-H mice [19]. In the present study, to further establish the relevance of decreased Willin/FRMD6 expression in AD, we analyzed Willin/*FRMD6* transcript levels in microarray and RNA-Seq gene expression datasets from AD patient [23,39] and mouse model brains [24,30]. Consistent with the previous report of Willin/*Frmd6* downregulation in AD mouse model cortices, we found significant downregulation of Willin/*Frmd6* transcripts in the hippocampi of AD mouse models, but at a lower magnitude than was observed in the cortices (Table 1). Similarly, in AD patient brains Willin/*FRMD6* transcripts were significantly reduced (Table 1). Taken together, these results demonstrate that there is a consistent decrease in Willin/*FRMD6* transcript expression in the brains of AD patients and mouse models compared to non-AD controls.

### 3.2. Aβ Downregulates Willin/FRMD6 Expression through Oxidative Stress

**Figure 1:**
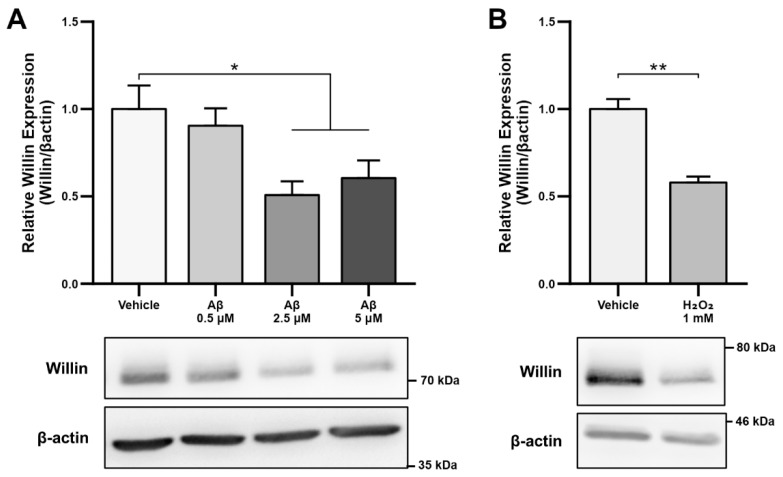

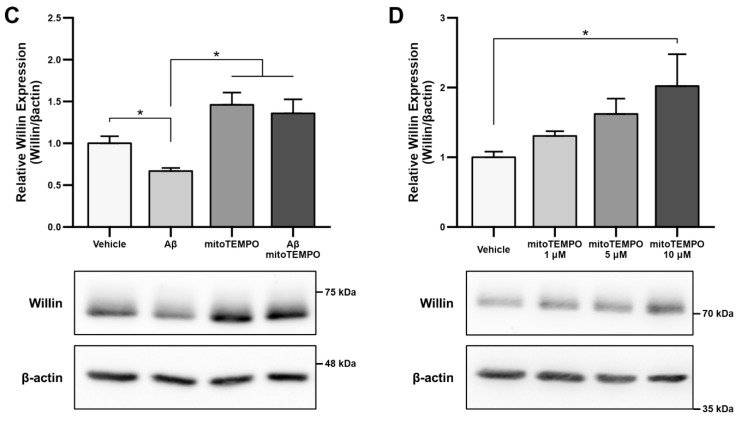
Aβ and oxidative stress downregulate Willin/FRMD6 expression in hippocampal HT-22 cells. Willin/FRMD6 protein levels in mouse hippocampal HT-22 cells after the following treatments: (**A**) Aβ at the indicated concentrations for 24 h, *n* = 4–6 per group; (**B**) H_2_O_2_ at 1 mM for 24 h, *n* = 3 per group; (**C**) MitoTEMPO, a MnSOD mimetic, pretreatment (10 μM) for 1 h prior to addition of Aβ (2.5 μM) for 24 h, *n* = 3 per group; (**D**) mitoTEMPO at the indicated concentrations for 24 h, *n* = 3 per group. β-actin served as a protein loading control. Upper panels represent quantification of immunoreactive bands relative to β-actin. Data are expressed as fold change relative to the vehicle control group. Representative immunoblots are shown underneath. * *p* < 0.05. ** *p* < 0.01.

As alterations in transcript levels may not correspond to protein level changes [41], we next investigated whether the reported downregulation of Willin/*FRMD6* transcripts seen in AD patient and mouse model RNA data translated to an Aβ-induced protein level dysregulation. Willin/FRMD6 expression was measured by immunoblot in mouse hippocampal HT-22 cells exposed to Aβ for 24 h. Dose-dependent downregulation of Willin/FRMD6 expression was observed following Aβ exposure, with significant decreases seen in cells exposed to 2.5 μM of Aβ (Figure 1A). MTT reduction assays confirmed that this dose of Aβ was in the sublethal range for HT-22 cells (Figure S1), indicating that reductions in Willin/FRMD6 expression are not due to increased cell death.

Since accumulation of reactive oxygen species (ROS) is also one of the key early pathologies associated with Aβ toxicity [42], we next examined whether Willin/FRMD6 expression is affected by ROS. Exposure of HT-22 cells to H_2_O_2_ for 24 h resulted in decreased Willin/FRMD6 expression (Figure 1B). Importantly, the treatment concentration represented a sublethal dose of H_2_O_2_ as shown by the lack of significant difference in MTT reduction in H_2_O_2_-treated versus vehicle-treated cells (Figure S2). Following this result, as mitochondria are primary sites of cellular ROS generation, we next evaluated whether scavenging mitochondrial-derived ROS could reverse Aβ-mediated decreases in Willin/FRMD6 expression. HT-22 cells were pre-treated with mitoTEMPO, a mitochondrialy-targeted superoxide dismutase mimetic [43], prior to exposure to Aβ. Cells pretreated with mitoTEMPO did not display decreased Willin/FRMD6 expression in response to Aβ exposure (Figure 1C). Consistent with the role of mitochondrialy derived ROS in modulating Willin/FRMD6 expression, treatment with mitoTEMPO alone resulted in increased Willin/FRMD6 (Figure 1D). These data indicate that AD relevant pathologies, specifically Aβ and oxidative stress, induce downregulation of Willin/FRMD6 expression.

### 3.3. Downregulation of Willin/FRMD6 Disrupts Mitochondrial Function

**Figure 2:**
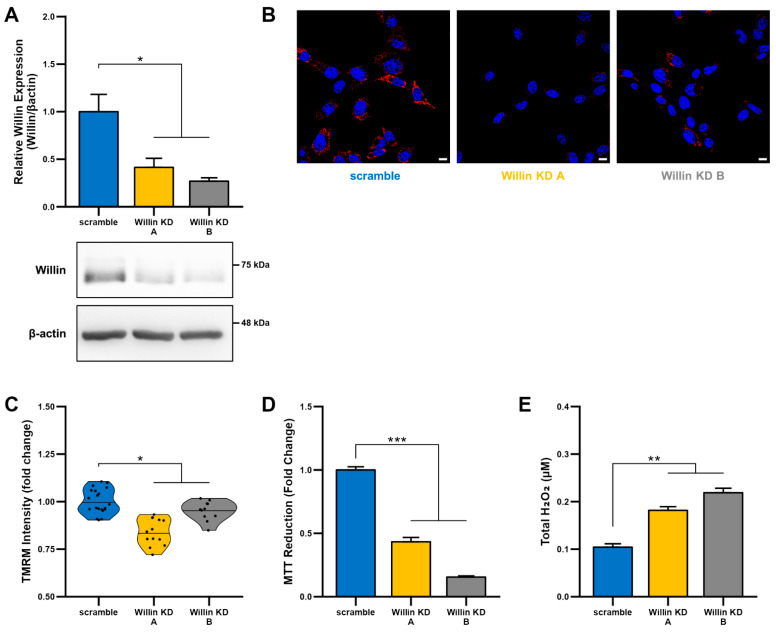
Downregulation of Willin/FRMD6 induces mitochondrial dysfunction in HT-22 cells. (**A**) Densitometry of Willin/FRMD6 immunoreactive bands in lysates from control (scramble) and Willin/FRMD6 knockdown (Willin KD A and Willin KD B) cells. β-actin served as a protein loading control. Upper panel represents quantification of immunoreactive bands relative to β-actin. Data are expressed as fold change relative to the control group. Representative immunoblots are shown underneath. *n* = 3 per group. * *p* < 0.05. (**B**,**C**) Mitochondrial membrane potential was measured by TMRM staining intensity. (**B**) Representative images of TMRM staining. Scale bar = 10 μm. (**C**) Quantification of mitochondrial staining intensity for TMRM using MitoSegNet. *n* = 11–20 fields of view (FOV) per condition with 10–50 cells per FOV. Dots represent mean TMRM staining intensity per FOV. * *p* < 0.05. (**D**) Mitochondrial redox capacity measured by MTT reduction assay. *n* = 3 per group. *** *p* < 0.001. (**E**) Total ROS production determined by Amplex^TM^ Red assay. *n* = 3 per group. ** *p* < 0.01.

To explore the functional consequences of reduced Willin/FRMD6 expression in neuronal cells, we generated HT-22 cell lines (Willin KD A and Willin KD B, collectively referred to as Willin KD) with reduced expression of endogenous Willin/FRMD6 using two short hairpin interference constructs and a control cell line (scramble) transduced with a scrambled short hairpin interference construct. Successful knockdown of Willin/FRMD6 expression was confirmed by immunoblotting (Figure 2A). Approximately 50% knockdown was achieved through our shRNA constructs.

Since mitochondrial function depends on the ability to maintain a healthy membrane potential, we first examined the effect of Willin/FRMD6 on mitochondrial membrane potential using the cell-permeant cationic dye, TMRM. This mitochondrial membrane permeant dye is readily taken up by active mitochondria and fluorescence intensity is higher in intact mitochondria. Willin KD cells displayed significantly decreased TMRM staining intensity compared to scramble cells (Figure 2B,C), suggesting that decreased Willin/FRMD6 expression promotes mitochondrial membrane depolarization.

Given that the mitochondrial membrane potential is critical for energy production by the electron transport chain (ETC), we next evaluated mitochondrial function/redox potential by MTT reduction. Consistent with the observation of loss of mitochondrial membrane potential in Willin KD cells, Willin KD cells also displayed significantly decreased MTT reduction capacity (Figure 2D) compared to scramble cells. Because increased oxidative stress and ROS deleteriously affect mitochondrial function [44], we tested whether Willin KD cells had abnormal accumulation of ROS by measuring H_2_O_2_ levels. Total H_2_O_2_ levels were significantly increased in Willin KD cells compared to scramble controls (Figure 2E). Taken together, these data indicate that Willin/FRMD6 knockdown decreases mitochondrial function leading to increased ROS production.

### 3.4. Willin/FRMD6 Knockdown Leads to Mitochondrial Fragmentation in HT-22 Cells

**Figure 3:**
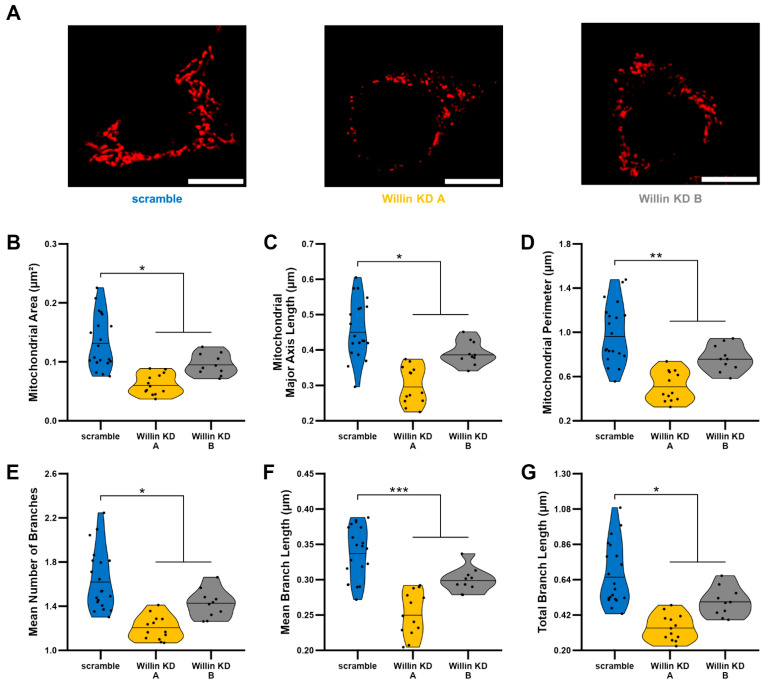
Willin/FRMD6 knockdown leads to mitochondrial fragmentation. Mitochondrial morphological and network abnormalities in Willin KD cells. HT-22 cells were labeled with TMRM for visualization of mitochondria morphology. (**A**) Representative images of TMRM staining. Scale bar = 10 μm. (**B**–**G**) Quantitative measurement of mitochondrial morphological (**B**–**D**) and network parameters (**E**–**G**) using MitoSegNet for segmentation and analysis. *n* = 10–20 fields of view (FOV) per condition with 10–50 cells per FOV. Dots represent means per FOV. * *p* < 0.05. ** *p* < 0.01. *** *p* < 0.001.

Because mitochondrial fission and fusion play critical roles in the maintenance of mitochondrial function [45,46], we next evaluated mitochondrial morphology using an unbiased deep-learning based segmentation and analysis tool, MitoSegNet [38]. Morphologically, mitochondria in Willin KD cells were more fragmented and punctate compared to scramble controls (Figure 3A). Willin KD cells displayed significant decreases in mitochondrial area (Figure 3B), mitochondrial length (Figure 3C), and mitochondrial perimeter (Figure 3D) along with mitochondrial network defects including decreased number of branches (Figure 3E), mean branch length (Figure 3F), and total branch length (Figure 3G) indicative of mitochondrial network fragmentation.

**Figure 4:**
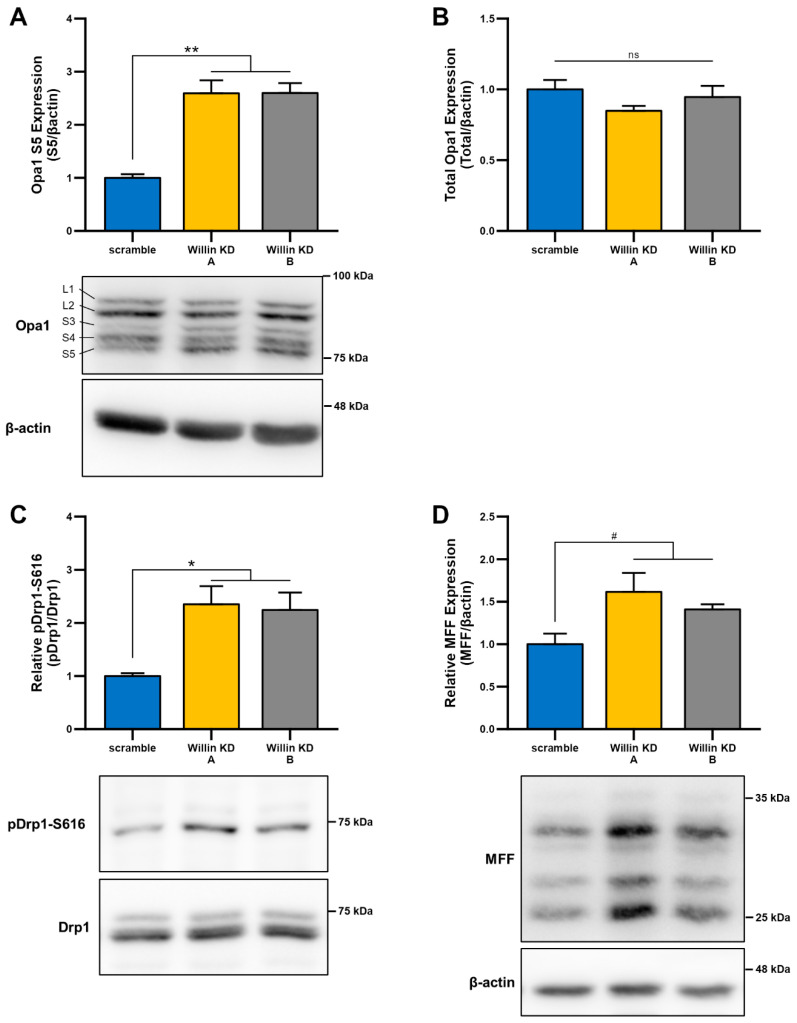
Downregulation of Willin/FRMD6 alters levels of mitochondrial fission and fusion proteins. (**A**,**B**) Quantification of OPA1 (S5) (**A**) and total OPA1 (**B**) immunoreactive bands relative to β-actin in the indicated cell lysates. Short isoforms of OPA1 are associated with mitochondrial fission. Data are expressed as fold change relative to the scramble group. *n* = 3 per group. ** *p* < 0.01. ns = no significant difference between all groups. (**C**) Quantification of phospho-DRP1-S616 immunoreactive bands normalized to total DRP1 in the indicated groups. Data are expressed as fold change relative to the scramble group. Representative immunoblots are shown underneath. *n* = 3 per group. * *p* < 0.05. (**D**) Quantification of MFF immunoreactive bands, normalized to β-actin in the indicated groups. Data are expressed as fold change relative to the scramble group. Representative immunoblots are shown underneath. *n* = 3 per group. # *p* < 0.1.

Given that, mitochondrial morphology is regulated through a careful balance between mitochondrial fusion and fission, which are governed by highly conserved mitochondrial dynamics proteins with GTPase function [47,48], we next examined whether Willin/FRMD6-induced changes in mitochondrial morphology were mediated by alterations in mitochondrial fission and fusion proteins. To explore whether impairment of mitochondrial fission and fusion are involved in the induction of mitochondrial fragmentation with Willin/FRMD6 knockdown in HT-22 cells, cell lysates were subjected to immunoblot analysis for levels of mitochondrial fission and fusion proteins (Figure 4). Willin KD cells displayed significant increases in short OPA1 (Figure 4A), without significant changes in total OPA1 levels (Figure 4B). This is indicative of dysregulated OPA1 processing that favors mitochondrial fission rather than fusion [49]. Consistently, DRP1 phosphorylation at S616 is increased in Willin KD cells compared to scramble controls (Figure 4C). Furthermore, there is a trend towards increased expression of mitochondrial fission factor (MFF) in Willin KD cells (Figure 4D). Phosphorylation of DRP1 at S616 and recruitment to the mitochondrial outer membrane by MFF is associated with ERK-signaling-mediated mitochondrial fission [50]. Thus, Willin/FRMD6 knockdown disrupts levels of mitochondrial fission and fusion proteins in a manner that favors mitochondrial fission and fragmentation.

### 3.5. Willin/FRMD6 Knockdown Induces Mitochondrial Dysfunction in Primary Neurons

**Figure 5:**
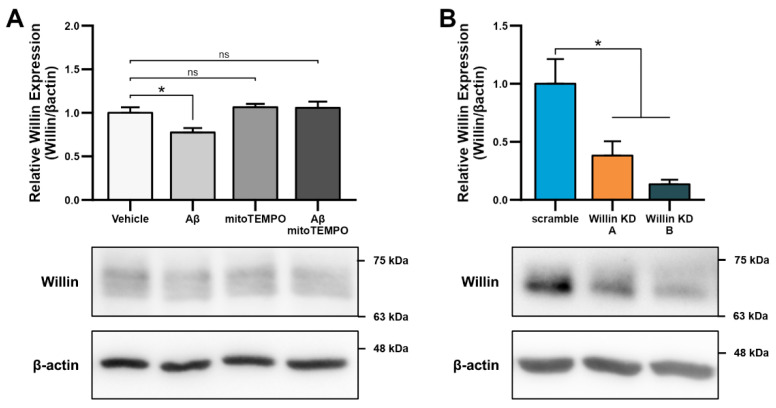

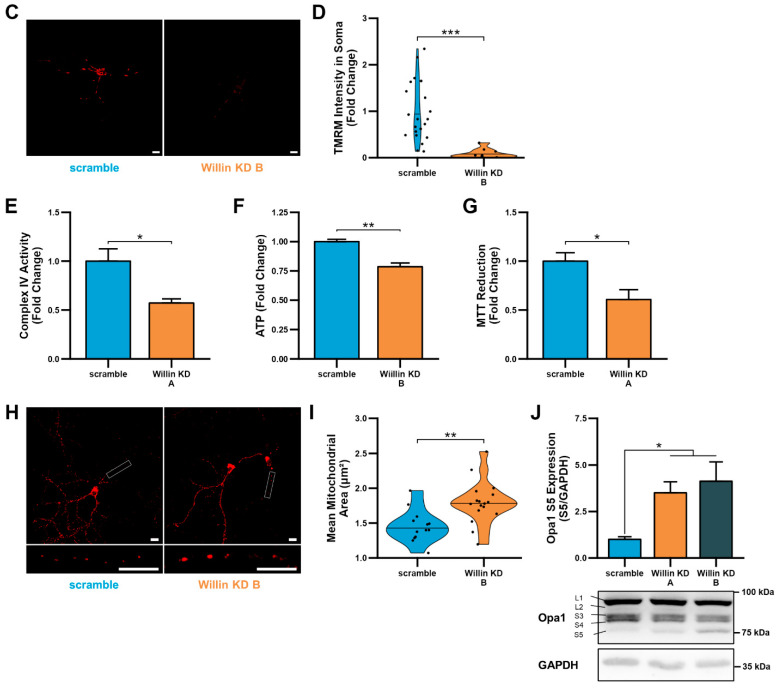
Downregulation of Willin/FRMD6 induces mitochondrial dysfunction in primary mouse neurons. (**A**) Primary mouse cortical neurons (DIV 10) were exposed to 0.5 μM Aβ in 0.5× B-27 for 24 h with or without 10 μM mitoTEMPO pretreatment (1 h) and then subjected to immunoblot analysis for Willin/FRMD6 expression. β-actin served as a protein loading control. Upper panel represents quantification of immunoreactive bands relative to β-actin. Data are expressed as fold change relative to the vehicle group. Representative immunoblots are shown underneath. *n* = 3 per group. * *p* < 0.05. ns = not significant. (**B**) Densitometry of Willin/FRMD6 immunoreactive bands in lysates from control (scramble) and Willin/FRMD6 knockdown (Willin KD A and Willin KD B) cortical neurons (DIV 15). β-actin served as a protein loading control. Upper panel represents quantification of immunoreactive bands relative to β-actin. Data are expressed as fold change relative to the scramble control group. Representative immunoblots are shown underneath. *n* = 4–5 per group. * *p* < 0.05. (**C**,**D**) Mitochondrial membrane potential was measured by TMRM staining intensity. (**C**) Representative images of TMRM staining in primary hippocampal neurons. Scale bar = 10 μm. (**D**) Quantification of soma staining intensity for TMRM using NIH ImageJ. *n* = 9–22 neurons per condition. Dots represent individual neurons. *** *p* < 0.001. (**E**) Enzymatic activity of cytochrome c oxidase (mitochondrial complex IV) was determined in cell lysates from cortical neurons from the indicated groups. *n* = 3 per group. * *p* < 0.05. (**F**) ATP levels in cortical neurons were measured by luciferase assay in the indicated groups. *n* = 4 per group. Data are expressed as fold change relative to the scramble group. ** *p* < 0.01. (**G**) Mitochondrial redox capacity measured by MTT reduction assay in cortical neurons. *n* = 6 per group. * *p* < 0.05. (**H**,**I**) Primary hippocampal neurons were labeled with anti-TOM20 for visualization of mitochondrial morphology. (**H**) Representative images of TOM20 staining. Scale bar = 10 μm. (**I**) Quantitative measurement of mitochondrial area using NIH ImageJ. *n* = 13–17 fields of view (FOV) per group with 36–314 mitochondria per FOV. Dots represent means per FOV. ** *p* < 0.01. (**J**) Quantification of OPA1 (S5) immunoreactive band relative to GAPDH in cortical neurons. Short isoforms of OPA1 are associated with mitochondrial fission. Data are expressed as fold change relative to the scramble group. Representative immunoblots with quantified band (S5) are shown underneath. *n* = 4–5 per group. * *p* < 0.05.

Because the cell-type specific nature of the consequences of altered Willin/FRMD6 expression have previously been noted [51] and because the effects of Willin/FRMD6 modulation in primary central nervous system neurons are presently unknown, we confirmed the role of Willin/FRMD6 in modulating mitochondrial function in primary mouse cortical and hippocampal neurons. Consistent with the results in HT-22 cells, exposure of primary neurons to Aβ resulted in decreased Willin/FRMD6 expression, which was ameliorated by pre-treatment with mitoTEMPO (Figure 5A). shRNA-mediated knockdown of Willin/FRMD6 expression in primary neurons resulted in a 60–90% reduction in Willin/FRMD6 expression (Figure 5B). While the banding pattern of Willin/FRMD6 differs between Figure 5A,B, the molecular weight of the immunoreactive band is the same in both blots. The difference may be due to differences in neuron age (DIV 10 in Figure 5A versus DIV 15 in Figure 5B) or culture conditions (Aβ treatment in growth media with reduced B-27 supplementation in Figure 5A versus normal growth media in Figure 5B) that could lead to variations in Willin/FRMD6 post-translational modifications, such as glycosylation.

Willin/FRMD6 knockdown neurons displayed significantly decreased mitochondrial membrane potential as shown by reduced TMRM staining intensity in the soma (Figure 5C,D). The reduction in mitochondrial membrane potential was associated with decreased mitochondrial function as shown by decreased complex IV activity, ATP production, and MTT reduction (Figure 5E–G).

Consistently, Willin/FRMD6 knockdown neurons also displayed abnormal mitochondrial morphology as shown by mitochondrial swelling in the processes of primary hippocampal neurons (Figure 5H,I). Willin/FRMD6 knockdown neurons also exhibited an increase in expression of short OPA1, without significant changes in total OPA1 levels (Figure 5J), suggesting that changes in mitochondrial morphology in primary neurons may result from dysfunctional OPA1 processing that prevents mitochondrial fusion-driven quality control leading to mitochondrial swelling. These results in primary neurons indicate that the role of Willin/FRMD6 in mediating mitochondrial function and dynamics extends to both mitotic and post-mitotic neuronal cells.

### 3.6. Knockdown of Willin/FRMD6 Triggers Aβ-Mediated Mitochondrial Alterations

**Figure 6:**
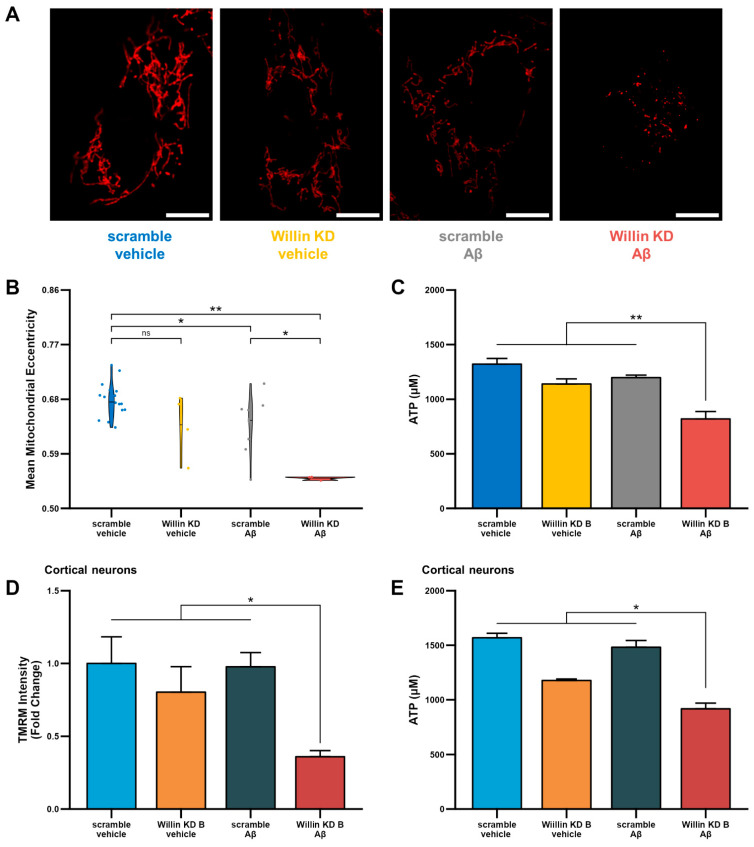
Downregulation of Willin/FRMD6 triggers Aβ-induced mitochondrial dysfunction in HT-22 cells and primary mouse neurons. (**A**,**B**) HT-22 cells were exposed to Aβ (5 μM) for 48 h prior to labeling with TMRM for visualization of mitochondria morphology. (**A**) Representative images of TMRM staining. Scale bar = 10 μm. (**B**) Quantitative measurement of mitochondrial eccentricity using MitoSegNet. *n* = 3–16 FOV per group with 2–28 cells per FOV. Dots represent means per FOV. * *p* < 0.05. ** *p* < 0.01. ns = not significant. (**C**) ATP levels in HT-22 cells were measured by luciferase assay following exposure to Aβ (10 μM) for 24 h. *n* = 3 per group. ** *p* < 0.01. (**D**) Mitochondrial membrane potential was measured by TMRM staining intensity in primary mouse cortical neurons following exposure to Aβ (1 μM) for 6 h by plate reader assay. Data are expressed as fold change relative to the vehicle-treated scramble control. *n* = 6 per group. * *p* < 0.05. (**E**) ATP levels in primary mouse cortical neurons were measured by luciferase assay following exposure to Aβ (0.5 μM) for 24 h. *n* = 3 per group. * *p* < 0.05.

Having shown that depletion of Willin/FRMD6 leads to mitochondrial dysfunction, we next investigated whether, Willin/FRMD6 knockdown enhances susceptibility to the deleterious effects of Aβ on mitochondrial function and structure. To do so, Willin KD and scramble controls were exposed to Aβ and changes in mitochondrial morphology and function were assessed. Morphological changes were assessed using MitoSegNet [38], which uses deep-learning based segmentation to return morphological parameters including mitochondrial area, eccentricity, aspect ratio, perimeter, and solidity. In HT-22 cells, Willin/FRMD6 knockdown exacerbated Aβ-induced decreases in mitochondrial eccentricity (Figure 6A,B). Decreased mitochondrial eccentricity is associated with decreased mitochondrial length and development of more punctate morphology. Consistent with these abnormalities in mitochondrial morphology, HT-22 cells with Willin/FRMD6 knockdown exposed to Aβ displayed significantly decreased ATP production compared to scramble controls exposed to Aβ (Figure 6C). Similarly, primary cortical neurons with depletion of Willin/FRMD6 exhibited significantly larger Aβ-induced reductions in TMRM staining intensity and ATP production compared to control neurons (Figure 6D,E). Taken together, these data indicate that Willin/FRMD6 knockdown increases vulnerability to Aβ-mediated mitochondrial dysfunction. Given that Aβ treatment alone reduces Willin/FRMD6 expression, these results demonstrate a potential feedback mechanism whereby Aβ-induced downregulation of Willin/FRMD6 expression further exaggerates mitochondrial defects associated with amyloid pathology.

### 3.7. Overexpression of Willin/FRMD6 Attenuates Aβ-Induced Toxicity

Given that Willin/FRMD6 knockdown promotes mitochondrial dysfunction and increases vulnerability to the deleterious effects of Aβ toxicity on mitochondrial function, we next asked whether overexpression of Willin/FRMD6 could ameliorate the detrimental effects of Aβ on mitochondrial function and structure. Using a retroviral construct, we generated a monoclonal HT-22 cell line with overexpression of Willin/FRMD6 and confirmed successful overexpression by immunoblot (Figure S3). To overexpress Willin/FRMD6 in primary neurons, we generated an adeno-associated viral vector and confirmed overexpression by immunoblot and immunofluorescence (Figure S4).

**Figure 7:**
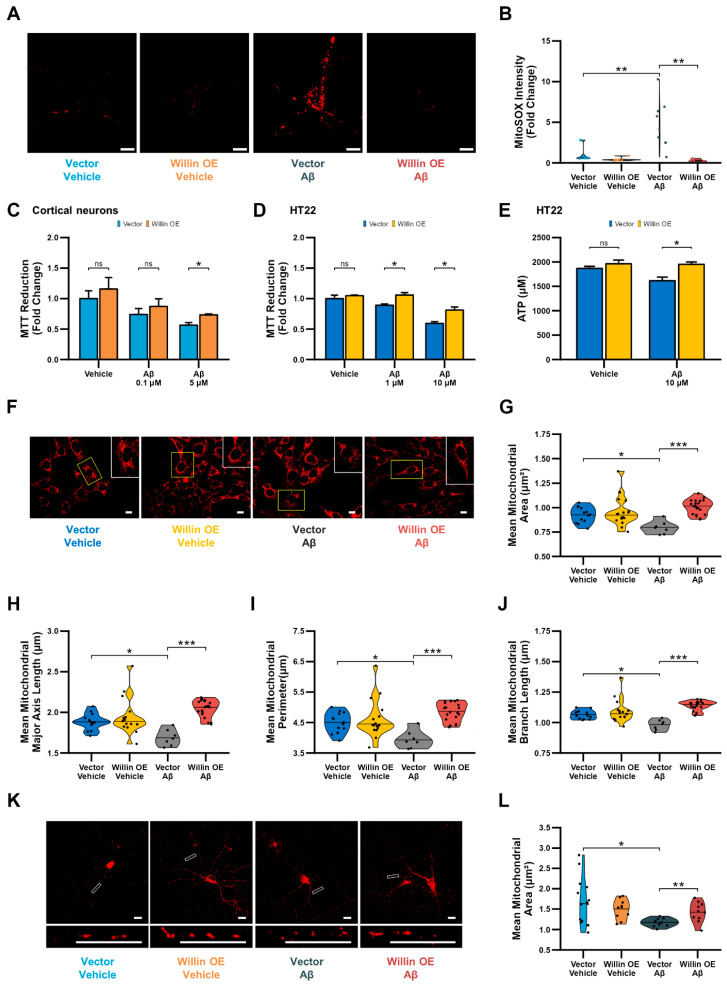
Overexpression of Willin/FRMD6 in mouse hippocampal HT-22 cells and primary mouse neurons attenuates Aβ-induced deficits in mitochondrial function and structure. (**A**,**B**) Primary hippocampal neurons were exposed to Aβ (0.5 μM) for 48 h before measurement of mitochondrial reactive oxygen species by MitoSOX Red staining intensity. (**A**) Representative images of MitoSOX staining intensity in hippocampal neurons. Scale bar = 10 μm. (**B**) Quantification of mitochondrial staining intensity for MitoSOX using NIH ImageJ, *n* = 6–9 FOV per condition with 1-5 neurons per FOV. Dots represent means per FOV. ** *p* < 0.01. (**C**,**D**) Mitochondrial redox capacity measured by MTT reduction assay in (**C**) cortical neurons and (**D**) HT-22 cells after 24 h treatment at the indicated Aβ concentrations. *n* = 3–6 per group. * *p* < 0.05. ns = not significant. (**E**) ATP levels in HT-22 cells were measured by luciferase assay following exposure to Aβ at the indicated concentrations for 24 h. *n* = 3 per group. * *p* < 0.05. ns = not significant. (**F**–**J**) HT-22 cells were exposed to Aβ (10 μM) for 24 h before labeling with MitoTracker Red CMXRos for visualization of mitochondrial morphology. (**F**) Representative images of MitoTracker Red CMXRos staining in HT-22 cells. Scale bar = 10 μm. Insets of regions of interest outlined in yellow shown in upper right corners. (**G**–**J**) Quantitative measurement of (**G**) mitochondrial area, (**H**) mitochondrial major axis length, (**I**) mitochondrial perimeter, and (**J**) mean mitochondrial branch length using MitoSegNet. *n* = 7–20 FOV per group with 10–50 cells per FOV. Dots represent means per FOV. * *p* < 0.05. *** *p* < 0.001. (**K**,**L**) Primary hippocampal neurons were exposed to Aβ (0.5 μM) for 24 h prior to labeling with anti-TOM20 for visualization of mitochondrial morphology. (**K**) Representative images of TOM20 staining in hippocampal neurons in the indicated groups. Insets shown at the bottom. Scale bars = 10 μm. (**L**) Quantification of mitochondrial area in neuronal processes using NIH Image J. *n* = 10–15 FOV per group with 23–334 mitochondria per FOV. Dots represent means per FOV. * *p* < 0.05. ** *p* < 0.01.

Overexpression of Willin/FRMD6 in primary neurons attenuated Aβ-induced perturbations in mitochondrial function including enhanced production of mitochondrial reactive oxygen species (Figure 7A,B) and decreases in MTT reduction capacity (Figure 7C). Similarly, overexpression in HT-22 cells rescued Aβ-induced decreases in MTT reduction capacity (Figure 7D) and ATP production (Figure 7E). Furthermore, overexpression of Willin/FRMD6 protected against Aβ-induced imbalance in mitochondrial dynamics in HT-22 cells as shown by the amelioration of alterations in mitochondrial morphological parameters including decreases in mitochondrial area, major axis length, and perimeter (Figure 7F–I) and mitochondrial network parameters such as mean branch length (Figure 7J), indicating that Willlin/FRMD6 overexpression protects against Aβ-induced mitochondrial fragmentation. Consistent with these results, overexpression of Willin/FRMD6 in primary hippocampal neurons also reversed Aβ-induced mitochondria fragmentation (Figure 7K,L). Taken together these findings indicate that increasing expression of Willin/FRMD6 protects against Aβ-induced defects in mitochondrial function and fission/fusion balance.

### 3.8. Willin/FRMD6 Modulates ERK Signaling in Primary Neurons

**Figure 8:**
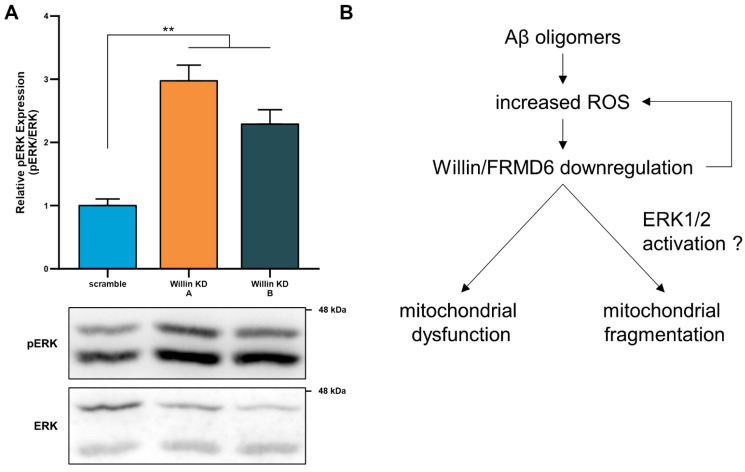
Willin/FRMD6 modulates a signaling pathway associated with neurodegeneration in primary neurons. (**A**) Quantification of phospho-p44/42 MAP Kinase (ERK1 and ERK2) immunoreactive bands relative to ERK1/2 in the indicated groups from primary cortical neuron lysates. Data are expressed as fold change relative to the scramble group. Representative immunoblots are shown underneath. *n* = 4–5 per group. ** *p* < 0.01. (**B**) Schematic diagram of the putative mechanism underlying Willin/FRMD6 involvement in AD pathogenesis. Aβ and amyloid pathology in AD increase mitochondrial ROS, leading to downregulation of Willin/FRMD6 expression, which leads to mitochondrial dysfunction and fragmentation in neuronal cells. These mitochondrial defects may be directly or indirectly mediated through Willin/FRMD6-induced activation of ERK signal transduction. Overexpression of Willin/FRMD6 rescues Aβ-induced mitochondrial perturbations.

ROS are strong stimulators for the activation of MAP kinases including those involved in ERK signal transduction. Recent studies [12] have demonstrated that Willin/FRMD6 is an upstream regulator of ERK signaling in an immortalized neuronal cell line, where reducing Willin/FRMD6 levels resulted in activation of ERK signaling; however, the signaling function of Willin/FRMD6 in primary neurons has yet to be reported. Immunoblot analysis of Willin/FRMD6-deficient primary neurons indicates that they exhibit significant increases in ERK phosphorylation (Figure 8A). ERK activation has been associated with mitochondrial dysfunction and enhanced ROS production in AD cybrid cells [16]. These data indicate that the potential mechanism underlying mitochondrial deficits associated with Willin/FRMD6 downregulation may involve activation of ERK signal transduction (Figure 8B).

## 4. Discussion

In the decade or so since Willin/*FRMD6* was identified as a potential AD risk gene by genome wide-association studies, the mechanisms underlying its potential role in AD pathogenesis have remained elusive. As oxidative stress and mitochondrial dysfunction are key early pathological features of AD [37,42,52,53,54,55], and are thus promising targets for the modulation/prevention of downstream neurodegeneration, in the present study, we addressed the key unexplored question of whether Willin/FRMD6 is involved in mitochondrial dysfunction in neurons and neuronal cell lines insulted by Aβ and oxidative stress.

First, we showed that there is significant down-regulation of Willin/*FRMD6* transcripts in AD mouse hippocampi and AD patient brains. These results are consistent with previous microarray studies that demonstrated significantly reduced Willin/FRMD6 transcripts in AD mouse cortices [19]. We extended these findings to the protein level by demonstrating that Aβ decreases Willin/*FRMD6* protein expression in neuronal cells through a potential mechanism involving oxidative stress and mitochondrial dysfunction. Future studies should further confirm whether the transcript level downregulation of Willin/*FRMD6* also occurs at the protein level in AD patient brains. As aging is a major risk factor for the development of AD, downregulation of Willin/FRMD6 in response to Aβ may represent a pathological facet of previous observations of decreased Willin/FRMD6 expression with cellular and organismal aging [11]. Interestingly, scavenging mitochondrial ROS by application of mitoTEMPO increased Willin/FRMD6 expression while H_2_O_2_ treatment decreased Willin/FRMD6 expression, suggesting that Willin/FRMD6 may act as a sensor for oxidative stress. Importantly, pretreatment with mitoTEMPO abrogated Aβ-induced decreases in Willin/FRMD6.

In primary cortical neurons exposed to Aβ with or without mitoTEMPO pre-treatment, we noted a difference in the appearance of the Willin/FRMD6 immunoreactive band (Figure 5A), though the molecular weight was the same as in other blots. Since the blots were processed under similar conditions, the difference may be due to post-translational modifications arising from differences in cell types (HT-22 versus primary cortical neurons), neuron culture age/maturation, and/or treatment conditions (reduced B-27 supplementation). Indeed, previous studies have shown that Willin/FRMD6 is involved in neuronal development [12] and that its expression differs between cell lines, cell passage, and organism age [11]; however, the specific mechanisms require further exploration.

In human AD brains and mouse models, mitochondrial morphology shifts towards excessive fission [54,56]. Our studies demonstrated that downregulation of Willin/FRMD6 in neuronal cells results in mitochondrial dysfunction and imbalanced mitochondrial dynamics. Morphologically, Willin/FRMD6 knockdown led to mitochondrial fragmentation in HT-22 cells and distinctive mitochondrial swelling in primary hippocampal neurons. This difference may be due to increased vulnerability of primary neurons to Willin/FRMD6-induced changes in mitochondrial structure, as mitochondrial swelling represents a more severe mitochondrial defect [57] that may occur as a result of mitochondrial permeability transition, which involves membrane depolarization, electron transport chain malfunction, and osmotic swelling ultimately leading to cell death [37]. As transmission electron microscopy (TEM) has been used to examine ultrastructural changes in mitochondria that are relevant to AD pathogenesis [21,58,59], future studies should consider the use of TEM to conduct a more detailed examination of mitochondrial morphological changes that result from depletion of Willin/FRMD6, particularly since the present study suggests that decreased Willin/FRMD6 affects processing of mitochondrial inner membrane protein OPA1 and thus may potentially affect the organization of the mitochondrial cristae. Furthermore, swollen mitochondria may impair mitochondrial respiration and organelle transport [60]; thus, whether Willin/FRMD6 affects mitochondrial trafficking warrants future investigation.

Mitochondria are dynamic organelles that engage in repeated cycles of fission and fusion that are critical for the maintenance of mitochondrial morphology, distribution, and function [61,62]. Defects in either mitochondrial fission or fusion lead to abnormal mitochondria distribution and cellular dysfunction [63,64], with neurons being particularly vulnerable given their high energy requirements and reliance on mitochondria for proper synaptic function [65]. Disrupted mitochondrial fission/fusion has been shown in AD brains, mouse models, and Aβ-treated cell cultures [66,67,68,69,70]. Indeed, our results indicate that Willin/FRMD6 knockdown in neuronal cells influences several components of the mitochondrial fission/fusion machinery, ultimately shifting the balance towards fission. HT-22 cells displayed increased phosphorylation of mitochondrial fission protein DRP1 at S616 with Willin/FRMD6 knockdown. Previous studies have shown that phosphorylation of DRP1 at S616 is significantly increased in AD brains and in primary hippocampal neurons following Aβ oligomer treatment [69]. Furthermore, we found that both primary neurons and HT-22 cells displayed increased expression of short OPA1 with Willin/FRMD6 knockdown. Accumulation of short cleavage forms of OPA1 is associated with mitochondrial fragmentation, apoptosis, and inhibition of mitochondrial fusion [49,71,72,73,74,75]. As short OPA1 arises from proteolytic cleavage stimulated by loss of mitochondrial membrane potential [75,76], our findings of Willin/FRMD6 knockdown-induced reductions in mitochondrial membrane potential suggest a potential mechanism underlying these observed changes in mitochondrial dynamics proteins. Given that mitochondrial fusion protects against mitochondrial dysfunction by facilitating mitochondrial content mixing thereby allowing for protein complementation, mtDNA repair, and redistribution of metabolites [77], mitochondrial dysfunction due to Willin/FRMD6 knockdown may arise due to decreased mitochondrial fusion owing to dysregulation of mitochondrial dynamics proteins.

While OPA1 regulates fusion of the mitochondrial inner membrane, fusion of the mitochondrial outer membrane is regulated by dynamin-related GTPases mitofusin 1 and 2 (MFN1 and 2) [60,78]. Levels of MFN1/2 are significantly decreased in AD patients [68,69] and AD cybrid cells [16]. Whether Willin/FRMD6 downregulation induces decreased mitochondrial fusion through interactions with MFN1/2 presents a promising avenue for future studies, particularly as Willin/FRMD6 and MFN2 appear to share functional roles. Mutations in MFN2 are associated with peripheral nerve degeneration in Charcot-Marie-Tooth disease type 2A [45], while Willin/FRMD6 is involved in peripheral nerve repair [10]. Moreover, recent studies have shown that both MFN2 and Willin/FRMD6 are involved in regulating the organization of the actin cytoskeleton, though these roles have yet to be established in primary neurons. *Mfn*2-null mouse embryonic fibroblasts display significant reduction in the amount of actin stress fibers [79]. Similarly, Willin/FRMD6 knockdown in MCF10A cells [15] and SH-SY5Y cells [12] results in impaired actin cytoskeleton organization. As the actin cytoskeleton is involved in the regulation of mitochondrial function and dynamics [80,81], the role of Willin/FRMD6 in mediating mitochondrial function and dynamics may occur through its modulation of the actin cytoskeleton and potential interactions with mitochondrial outer membrane proteins such as MFN2.

Phosphorylation of DRP1 at S616 and recruitment to the mitochondrial outer membrane by MFF is associated with ERK-signaling-mediated mitochondrial fission [50]. Importantly, we report for the first time the effects of manipulating Willin/FRMD6 expression in primary cortical neurons from the central nervous system. In primary cortical neurons, knockdown of Willin/FRMD6 results in activation of ERK1/2, which extends the findings of previous studies demonstrating Willin/FRMD6 regulation of ERK signaling in neuronal SH-SY5Y cells [12] to primary neurons. ERK activation is associated with increased oxidative stress and mitochondrial and neuronal stress [82,83,84] as well as mitochondrial fragmentation in AD cybrid cells [16]. Our results indicated that knockdown of Willin/FRMD6 in primary neurons results in activation of ERK1/2 and mitochondrial functional and morphological abnormalities, suggesting that Willin/FRMD6 knockdown may induce mitochondrial alterations through modulation of ERK signaling (Figure 8B); however, further studies are needed to elucidate the details. Interestingly, ScanSitePlus prediction of sequence motifs relevant to cellular signaling reveals that the human Willin/FRMD6 protein sequence contains predicted binding sites for ERK1 and ERK D-domains as well as AMPK substrate motifs [85]. AMPK mediates mitochondrial fission in response to cellular energy stress through phosphorylation of DRP1 at S616 [50]. Thus, it appears that Willin/FRMD6 may serve as a crossroads for signaling pathways associated with oxidative and energy stress and mitochondrial function.

The effect of Willin/FRMD6 on mitochondria may also be mediated through its role as an upstream regulator of Hippo signaling, which has been shown to influence mitochondrial structure and function in *Drosophila*. Specifically, downstream Hippo component *Yki* (YAP1/2 homolog) induces upregulation of *opa1* [86], while upstream Hippo component *Fat* is capable of direct binding to mitochondrial complex I [87]. Similarly, mammalian YAP1/2 has been shown to affect mitochondrial network fission/fusion, mitochondrial membrane potential and levels of DRP1 and MFN2 in differentiating myoblasts [88]. The relative contributions of ERK and/or Hippo signaling pathways to the effect of Willin/FRMD6 on mitochondrial function and dynamics presents a promising avenue for future investigations.

Previous studies have shown that downregulation of Willin/FRMD6 primes cells for neuronal differentiation [12]. Here, we demonstrate that Willin/FRMD6 knockdown in neuronal cells results in decreased mitochondrial membrane potential and increased ROS production. As the process of neuronal differentiation involves decreases in mitochondrial membrane potential [89] and increased intracellular ROS [90,91], these same effects may prove to be deleterious in an environment with chronic elevation of oxidative and mitochondrial stress, as occurs in AD. That is, knockdown of Willin/FRMD6 may result in cellular and mitochondrial alterations that both prime mitotic cells for differentiation and increase susceptibility of post-mitotic mature neurons to toxic insults. For example, loss of mitochondrial membrane potential renders damaged mitochondria incapable of fusion-mediated repair, a process which requires the inner mitochondrial membrane potential [92,93] and increased oxidative stress has a detrimental effect on mitochondrial dynamics [16,94]. In line with this, we observed that downregulation of Willin/FRMD6 triggered several aspects of Aβ-induced mitochondrial dysfunction including reductions in mitochondrial membrane potential and ATP production along with increased mitochondrial fragmentation.

Lastly, we demonstrated that overexpression of Willin/FRMD6 in both mitotic and post-mitotic neuronal cells rescues Aβ-induced deficits in mitochondrial function and morphology including increased mitochondrial ROS production, decreased MTT reduction, decreased ATP production, and mitochondrial fragmentation. Future studies may consider the use of TEM to delineate which Aβ-induced ultrastructural changes in mitochondria are ameliorated by overexpression of Willin/FRMD6.

Taken together, our results indicate that Willin/FRMD6 plays a critical role in mitochondrial fission/fusion balance and mitochondrial function. Our results suggest a potential mechanism where Aβ and oxidative stress induce downregulation of Willin/FRMD6, leading to mitochondrial dysfunction and fragmentation along with further increases in ROS production, forming a vicious cycle that exacerbates neuronal degeneration (Figure 8B). Importantly, we demonstrated that this process can be corrected by overexpression of Willin/FRMD6. Overall, these studies are the first to demonstrate that Willin/FRMD6 can affect mitochondrial structure and function and furthermore that expression of Willin/FRMD6 is altered by Aβ and oxidative stress; thus, we provide novel avenues for future investigation into the role of Willin/FRMD6 as an AD risk gene.

## Figures and Tables

**Table 1:** Willin/*FRMD6* expression in microarray and RNA-Seq datasets from AD patients and AD mouse models.

Tissue	Species	Dataset	logFC	*p* Value	P adj	Analysis
AD temporal lobe	Human	Microarray [40]	−1.06	--	--	[19]
AD frontal lobe	Human	Microarray [40]	−1.06	--	--	[19]
AD brain	Human	RNA-Seq [39]	−1.49	0.0001	0.0028	This study
AD medial temporal gyrus	Human	Microarray [23]	−0.87	0.0119	0.0536	This study
APP^NL-G-F/NL-G-F^ cortex, 12 months	Mouse	Microarray [19]	−1.22	<0.05	--	[19]
3×Tg-AD-H cortex, 12 months	Mouse	Microarray [19]	−1.26	<0.05	--	[19]
TPM hippocampus, 8 months	Mouse	RNA-Seq [30]	−0.49	0.01757	N/A	This study
TAU hippocampus, 8 months	Mouse	RNA-Seq [30]	−0.44	0.01667	N/A	This study
HO hippocampus, 8 months	Mouse	RNA-Seq [30]	−0.59	0.00207	N/A	This study
HO hippocampus, 8 months	Mouse	Microarray [24]	−0.346	0.041	0.166	This study

logFC = log2 fold change relative to non-AD control samples. P adj = *p* value after adjustment for multiple-testing. N/A = not applied. -- = information not provided by original study. APP^NL-G-F/NL-G-F^ = *APP* Swedish, Iberian, Arctic. 3×Tg-AD-H = *APP* Swedish, *MAPT* P301L, *PSEN1* M146V. TPM = *PSEN1* M146V. TAU = *MAPT* P301L. HO = *APP* Swedish, *PSEN1* M146V.

## Data Availability

The data presented in this study are available on request from the corresponding author.

## Supplementary Materials

The following supporting information can be downloaded at: https://www.mdpi.com/article/10.3390/cells11193140/s1, Supplementary Figure S1. MTT reduction assay in HT-22 cells exposed to the indicated concentrations of Aβ. Supplementary Figure S2. MTT reduction assay in HT-22 cells exposed to the indicated concentration of H_2_O_2_. Supplementary Figure S3. Overexpression of Willin/FRMD6 in mouse hippocampal HT-22 cells. Supplementary Figure S4. Overexpression of Willin/FRMD6 in primary mouse neurons.
