# Supplementary figures and images for: Willin/FRMD6 Mediates Mitochondrial Dysfunction Relevant to Neuronal Aβ Toxicity
Source: Cells. 2022 Oct 6;11(19):3140. doi: 10.3390/cells11193140 (PMC9562665; doi:10.3390/cells11193140)

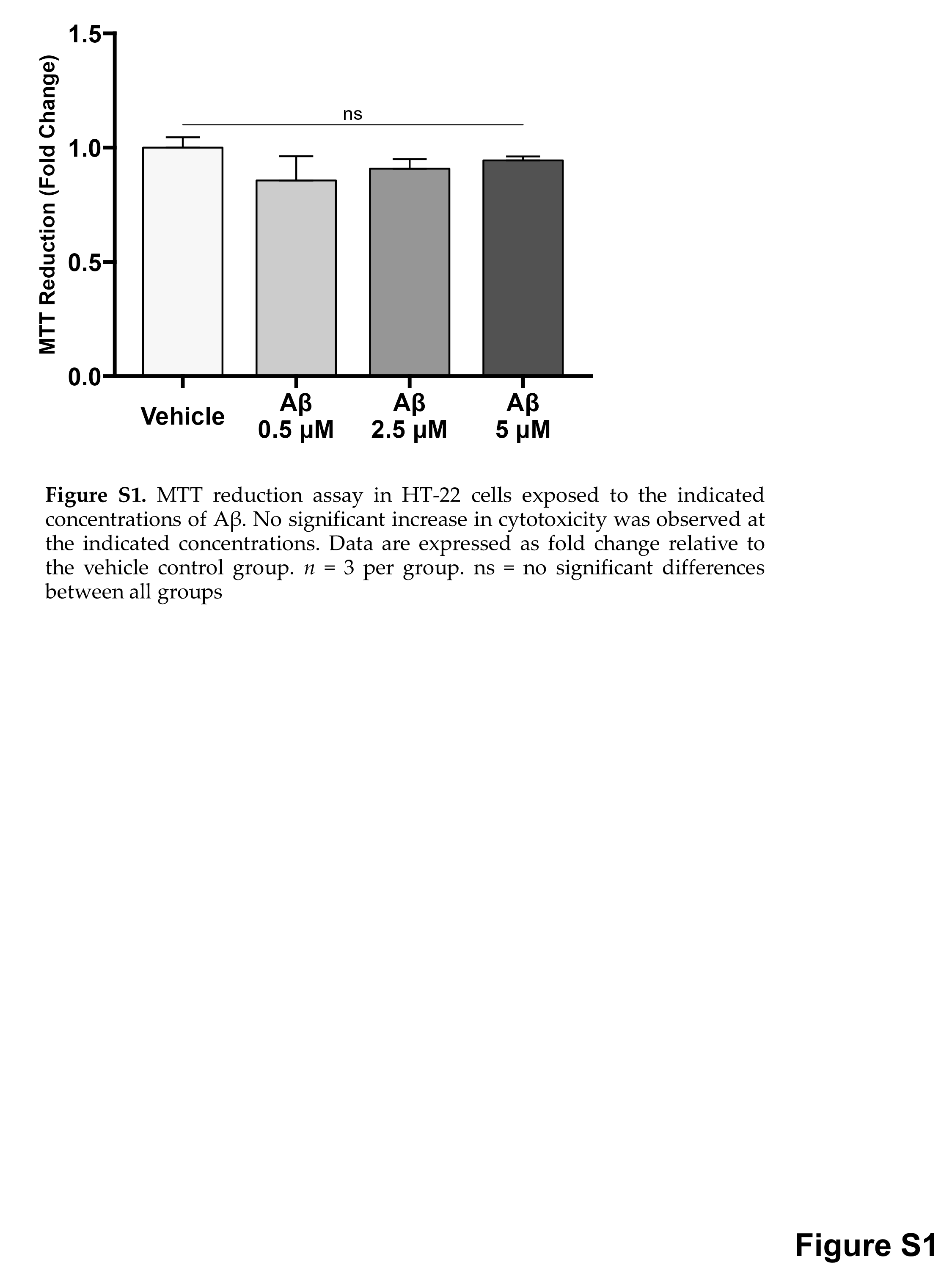

Supplement: Supplementary file 1 [file cells-11-03140-s001.zip › Supplementary/FigureS1.TIF]

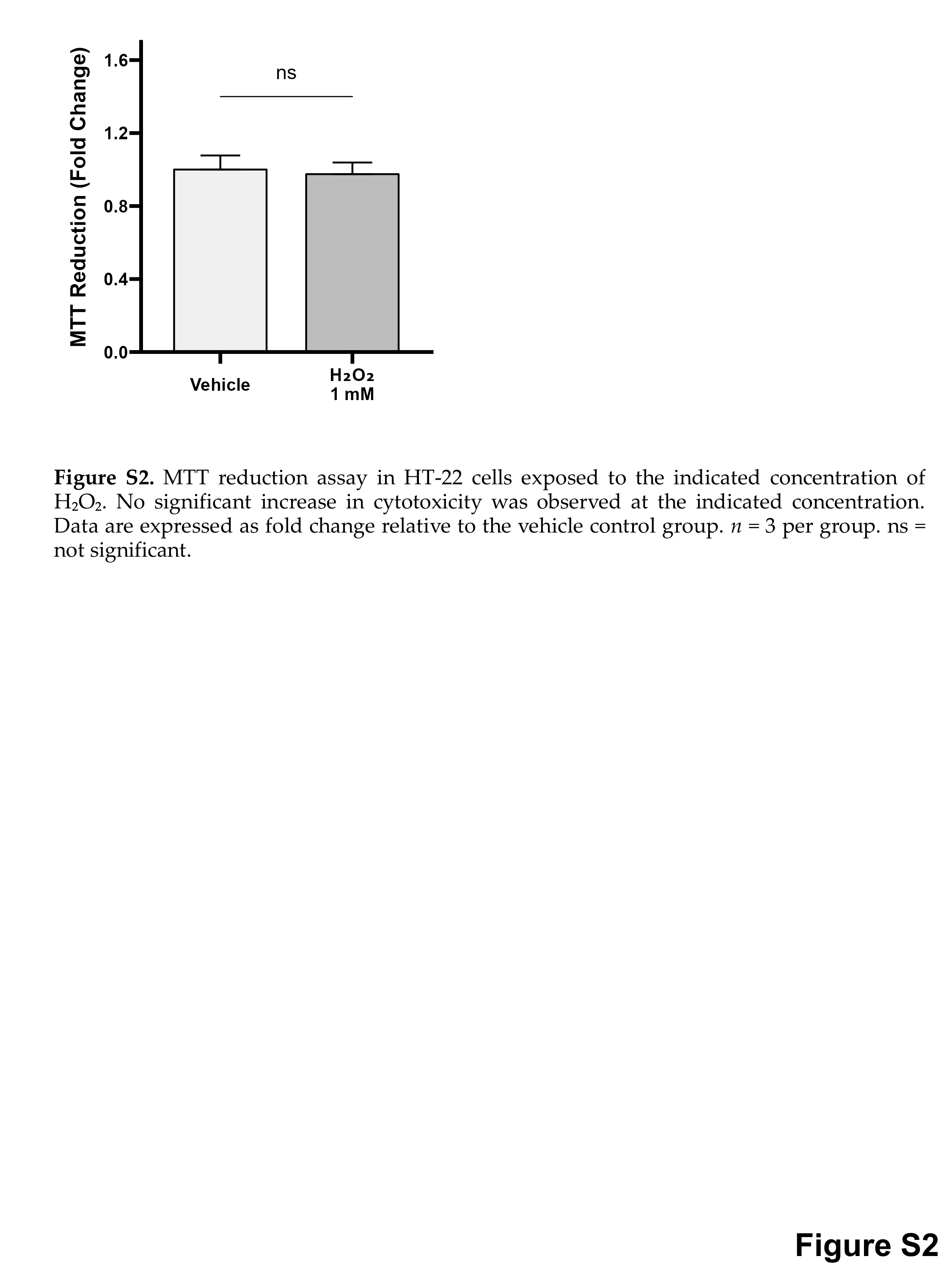

Supplement: Supplementary file 1 [file cells-11-03140-s001.zip › Supplementary/FigureS2.TIF]

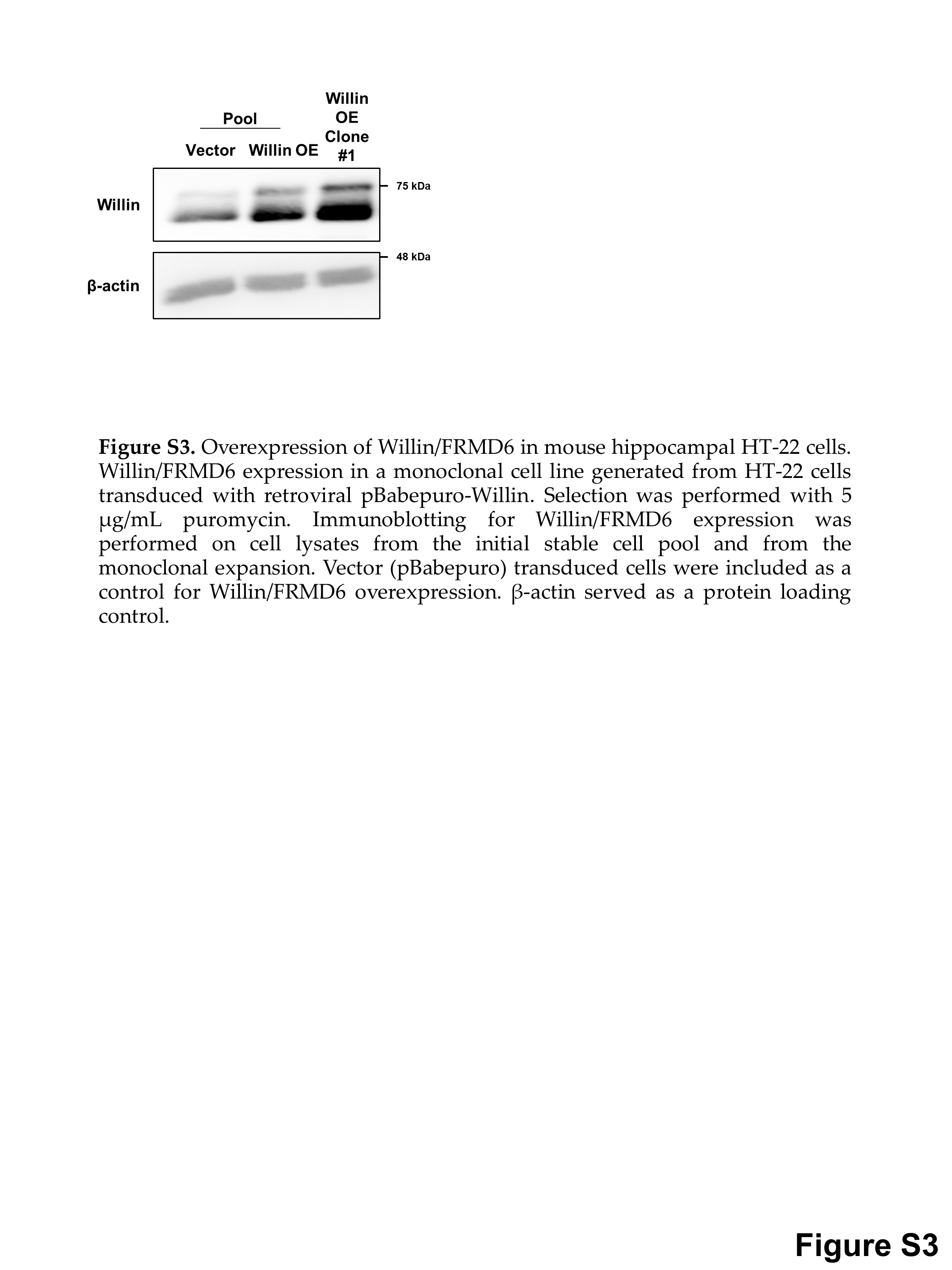

Supplement: Supplementary file 1 [file cells-11-03140-s001.zip › Supplementary/FigureS3.TIF]

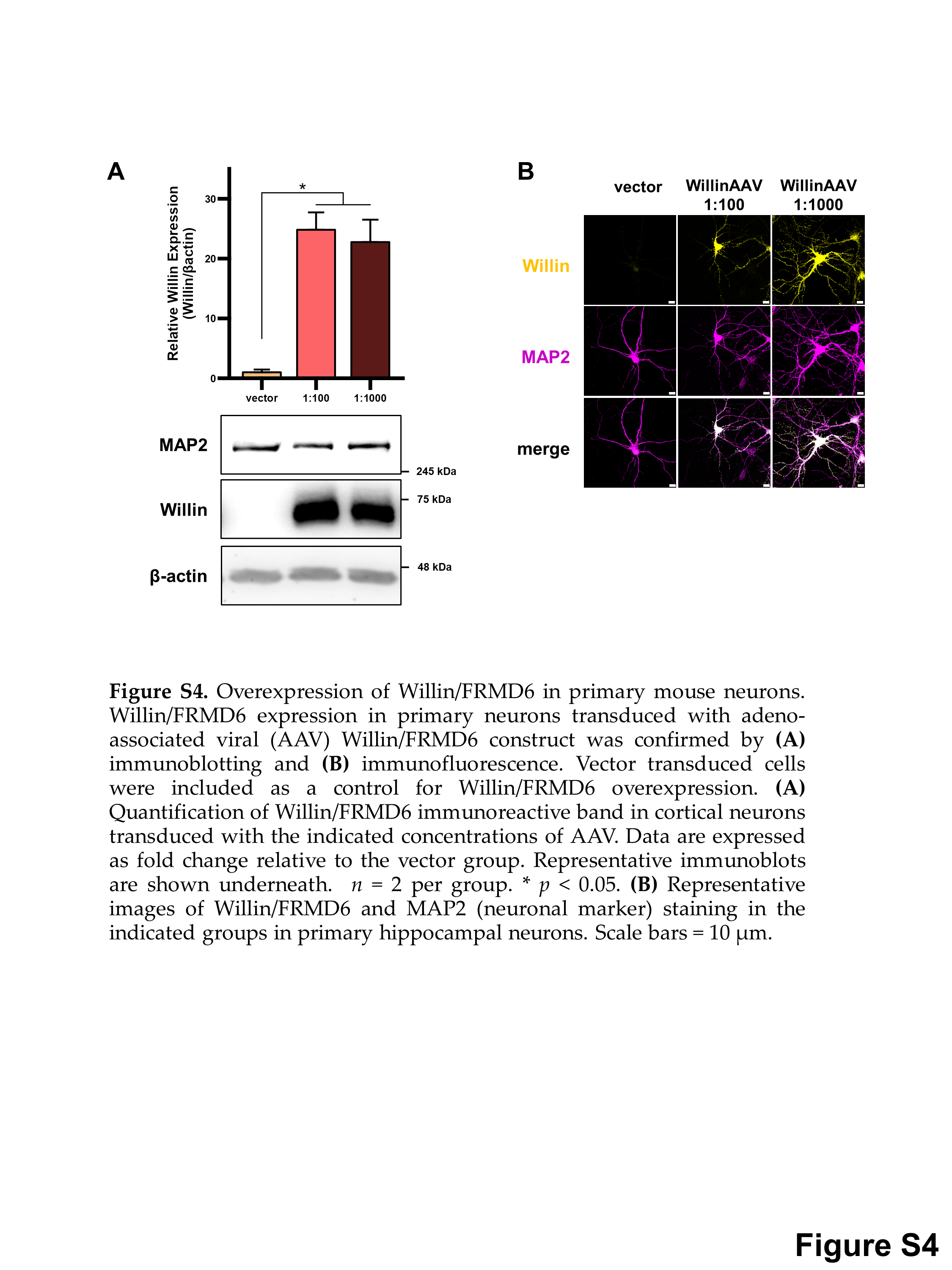

Supplement: Supplementary file 1 [file cells-11-03140-s001.zip › Supplementary/FigureS4.TIF]
